# Moderate and Severe Congenital Heart Diseases Adversely Affect the Growth of Children in Italy: A Retrospective Monocentric Study

**DOI:** 10.3390/nu15030484

**Published:** 2023-01-17

**Authors:** Daniela Palleri, Ylenia Bartolacelli, Anna Balducci, Simone Bonetti, Rossana Zanoni, Cristina Ciuca, Valentina Gesuete, Ambra Bulgarelli, Tammam Hasan, Luca Ragni, Emanuela Angeli, Gaetano Domenico Gargiulo, Andrea Donti

**Affiliations:** 1 Pediatric Cardiology and Adult Congenital Heart Disease Program, Department of Cardio-Thoracic and Vascular Medicine, IRCCS Azienda Ospedaliero-Universitaria di Bologna, Policlinico Sant’Orsola-Malpighi, 40138 Bologna, Italy; 2 Pediatric Cardiac Surgery and Adult Congenital Unit, Department of Cardio-Thoracic and Vascular Medicine, IRCCS Azienda Ospedaliero-Universitaria di Bologna, Policlinico Sant’Orsola-Malpighi, 40138 Bologna, Italy

**Keywords:** growth failure, children, malnutrition, pediatric heart diseases, congenital heart diseases, obesity

## Abstract

Children with congenital heart disease (CHD) are at increased risk for undernutrition. The aim of our study was to describe the growth parameters of Italian children with CHD compared to healthy children. We performed a cross-sectional study collecting the anthropometric data of pediatric patients with CHD and healthy controls. WHO and Italian z-scores for weight for age (WZ), length/height for age (HZ), weight for height (WHZ) and body mass index (BMIZ) were collected. A total of 657 patients (566 with CHD and 91 healthy controls) were enrolled: 255 had mild CHD, 223 had moderate CHD and 88 had severe CHD. Compared to CHD patients, healthy children were younger (age: 7.5 ± 5.4 vs. 5.6 ± 4.3 years, *p* = 0.0009), taller/longer (HZ: 0.14 ± 1.41 vs. 0.62 ± 1.20, *p* < 0.002) and heavier (WZ: −0,07 ± 1.32 vs. 0.31 ± 1.13, *p* = 0.009) with no significant differences in BMIZ (−0,14 ± 1.24 vs. –0.07 ± 1.13, *p* = 0.64) and WHZ (0.05 ± 1.47 vs. 0.43 ± 1.07, *p* = 0.1187). Moderate and severe CHD patients presented lower z-scores at any age, with a more remarkable difference in children younger than 2 years (WZ) and older than 5 years (HZ, WZ and BMIZ). Stunting and underweight were significantly more present in children affected by CHD (*p* < 0.01). In conclusion, CHD negatively affects the growth of children based on the severity of the disease, even in a high-income country, resulting in a significant percentage of undernutrition in this population.

## 1. Introduction

Children with congenital heart disease (CHD) are at greater risk for failure to thrive and are considered part of a nutritional high-risk group [1,2,3,4,5]. Undernutrition can manifest in different forms, namely wasting, stunting, underweight and micronutrient deficiencies. Wasting, stunting and underweight are defined as a child’s weight for height (or BMI), height and weight, respectively, being more than two standard deviations below the median for the international reference population of the same age. The reasons for undernutrition in children with CHD are many and of different nature, including hemodynamic instability [6,7], inadequate nutritional intake due to feeding fatigue [8,9], poor absorption of nutrients, fluid restriction, increased metabolic demands secondary to CHD physiopathology, heart failure and polycythemia due to chronic hypoxia [3]. Malnutrition entails loss of lean mass, including those of the heart and respiratory muscles, which compromise the myocardial and ventilatory functions. The healing capacity and immunological competency are also affected, with an increased risk of infections [10]. Together with reduced adult height and weight, poor growth also affects neurodevelopmental outcomes, with poorer school performance and reduced intellectual achievements [7,11,12,13].

Evidence of the negative influence of CHD on the development of infants and children comes mainly from low-income countries, where malnutrition is a social problem, and where corrective heart surgery often occurs later in life [1,14,15,16,17,18].

In some experiences from high-income countries, advancements in pediatric supportive cardiac care (use of increased calorie formulas and feeding tubes), early diagnosis and precocious surgery [19,20,21] have reduced but not removed the impact of CHD on nutritional status [22,23,24,25].

If young children with CHD are at risk of impaired growth [3,26], a shift toward overweight and obesity is reported in teenagers with CHD, similar to the general population [27,28,29,30,31,32,33,34]. In the cardiopathic population, restriction of physical activity for potential exercise intolerance, together with unhealthy eating habits, can increase the risk of childhood obesity and the resulting associated cardiovascular complications in adulthood.

The aim of this study was to define the impact of CHD on the growth of infants and children in a European high- income country, comparing the anthropometric parameters of patients with CHD to healthy children, with a special focus on the prevalence of wasting/underweight/stunting/overweight and obesity. Analyzing the characteristics of patients with the greatest difference in growth parameters can help to identify the critical periods for poor growth and obesity development and the right timing for possible intervention to improve the general well-being of patients with CHD and reduce their cardiovascular risk.

## 2. Materials and Methods

We performed a comparative retrospective cross-sectional study including all consecutive pediatric patients (1 month–18 years) attending the pediatric cardiology outpatient clinic from 1 January to 30 April 2021. Patients with genetic disease and other comorbidities known to affect growth (e.g., Down syndrome, DiGeorge syndrome, RASopathies, VACTERL/VATER association, short bowel syndrome, etc.) were excluded. For patients with more than one visit during the study period, the anthropometric measures at first evaluation were employed. The measures were collected at least 3 months after an eventual surgical intervention.

Our primary endpoint was to highlight any differences in growth parameters among patients affected by CHD, based on severity. From each patient, we collected the following information: height, weight, gender, age, comorbidities, cardiac diagnosis, history of pre-term birth or low birth weight, surgical history, number of previous surgical and percutaneous procedures, functional status, presence of cyanosis and oxygen saturation.

Anthropometric data were normalized for age and gender based on z-scores [35] for weight for age (WZ), length/height for age (HZ), weight for height (WHZ) in children younger than 2 years and body mass index (BMIZ) in children older than 2 years of age. World Health Organization tables were used for children aged < 2 years while Italian growth charts were used for children aged ≥ 2 years. According to World Health Organization, wasting was defined as WHZ ≤ –2 SD or BMIZ ≤ –2 SD, stunting was defined as HZ ≤ –2 SD and underweight was defined as WZ ≤ –2 SD. Overweight was defined as WZ > 2 SD, obesity was defined as WHZ > 3 SD in children younger than 2 years and BMIZ > 2 SD in older children; tall stature was defined as a HZ > 2 SD.

The patients were categorized by age and they were placed in one of the following groups: age ≤ 2 years, age > 2–5 years, age ≥ 5–18 years.

First, we divided patients in two groups according to the presence or absence of CHD.

The control group consisted primarily of patients referred to the cardiology outpatient’s clinic for chest pain, palpitations, heart murmurs or family history of heart disease, in which no organic heart disease was detected. The CHD patients were further divided according to the severity of heart disease—mild, moderate and severe, according to ESC Guidelines for CHD [36].

Mild disease included isolated congenital aortic or mitral valve disease (except parachute mitral valve and cleft), mild isolated pulmonary stenosis, isolated atrial septal defect (ASD), ventricular septal defect (VSD), and patent ductus arteriosus (PDA).

Moderate disease included anomalous pulmonary venous connection (partial or total), anomalous coronary arteries, subvalvular/supravalvuar aortic stenosis, atrioventricular septal defect (AVSD)—partial or complete (including primum ASD), coarctation of the aorta, Ebstein anomaly, tetralogy of Fallot repaired, transposition of the great arteries after arterial switch operation, VSD with associated anomalies or moderate or greater shunt, ASD with moderate or greater shunt, and moderate/severe pulmonary stenosis.

Severe disease included any cyanotic CHD (unoperated or palliated), univentricular circulation, double outlet right ventricle, pulmonary atresia, interrupted aortic arch, transposition of great arteries (except those that underwent arterial switch operation), and truncus arteriosus.

### Statistical Analysis

Data are expressed as mean ± standard deviation for continuous variables and number and percentage (%) for categorical variables. Differences in categorical variables were assessed with a chi-square test while differences in continuous variables were evaluated using a *t*-test or ANOVA with the Bonferroni test when needed.

Univariate and bivariate linear regression analyses were performed to assess the influence of CHD severity class and other risk factors on growth variables.

A *p*-value of <0.05 defined statistical significance.

Data were analyzed using STATA 15.1 (Copyright 1985–2017 StataCorp LLC, College Station, TX, USA).

## 3. Results

We enrolled 657 patients (566 with CHD and 91 healthy children). No difference in sex prevalence was noticed (53% males in healthy children vs. 54% males in CHD group, *p* = 0.812). Healthy patients were younger when compared to CHD patients (mean age: 5.6 ± 4.3 years vs. 7.5 ± 5.4 years, *p* = 0.001). In the CHD group, 255 (45%) children presented mild disease, 223 (39%) presented moderate disease and 88 (16%) presented severe CHD. Similar age distribution and male prevalence were noticed stratifying patients according to heart disease severity.

When compared to CHD patients, healthy children were slightly taller (mean HZ: 0.62 ± 1.20 vs. 0.14 ± 1.41, *p* < 0.002) and heavier (mean WZ: 0.31 ± 1.13 vs. −0.07 ± 1.32, *p* = 0.009), with no significant differences in BMI and WH (mean BMIZ: –0.07 ± 1.13 vs. −0,14 ± 1.24, *p* = 0.64; mean WHZ: 0.43 ± 1.07 vs. −0.05 ± 1.47, *p* = 0.1187).

Stunting was observed in 1.1% of healthy children versus 6.5% of CHD patients (*p* = 0.039); none of the healthy children presented underweight vs. 6.8% of CHD patients who did (*p* = 0.01) and wasting was detected in 2.2% of healthy children vs. 7.4% of CHD patients (*p* = 0.32 for BMIZ; *p* = 0.059 for WHZ).

On the other hand, no significant differences in overweight and obesity prevalence were noticed between CHD patients versus healthy children.

Focusing on CHD severity groups, a difference in nutritional status was detected, with moderate and severe CHD presenting lower mean HZ, WZ, BMIZ and WHZ. Stunting and underweight were more frequent in moderate and severe CHD, while wasting was more represented in children with severe CHD and in the first two years of life. Obesity was rare in all groups and similarly distributed. Tall stature was more frequent in healthy children and patients with mild CHD, compared to patients with moderate and severe CHD (Table 1).

Patients with severe CHD exhibited significantly lower mean WZ and HZ as detected by post-hoc Bonferroni analysis (WZ and HZ differences between healthy and severe CHD results in a *p* < 0.001); BMIZ and WHZ differences between severe CHD and healthy children resulted in *p* = 0.05 and *p* = 0.337, respectively.

Stratification in age groups revealed a significant lower WZ in CHD children younger than 5 years of age when compared to healthy children, and lower HZ in the group of cardiopathic children aged 2 to 5 years old. No relevant differences in BMIZ and WHZ were observed between the different age groups (Table 2).

In Table 3, a focus on the anthropometric parameters in the different age groups stratified for CHD severity is reported.

**Figure 1:**
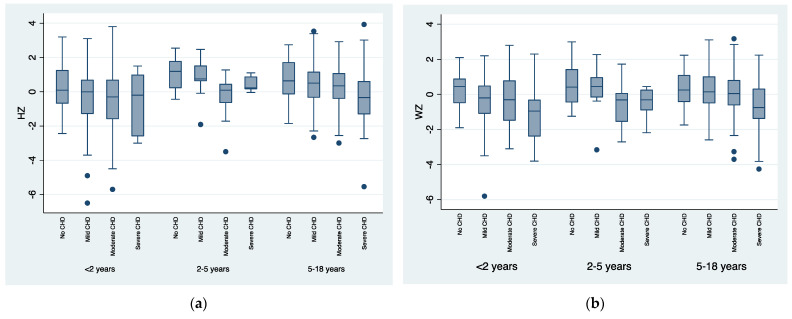

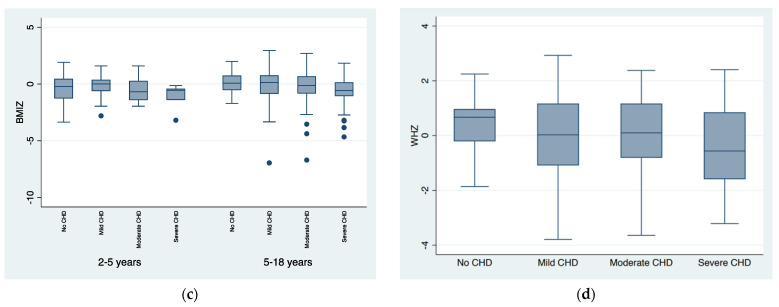
Box plots of length/height-for-age z-score (HZ), weight-for-age z-score (WZ), Body-Mass-Index-for-age z-score (BMIZ) and weight-for-height z-score (WHZ), stratified for the different ages and Congenital Heart Disease (CHD) severity groups. (**a**) Box plot of HZ distribution according to the different age groups and CHD severity groups; (**b**) Box plot of WZ distribution according to the different age groups and CHD severity groups; (**c**) Box plot of BMIZ distribution according to CHD severity groups in children older than 2 years of age; (**d**) Box plot of WHZ distribution according to CHD severity groups in children younger than 2 years of age.

**Figure 2:**
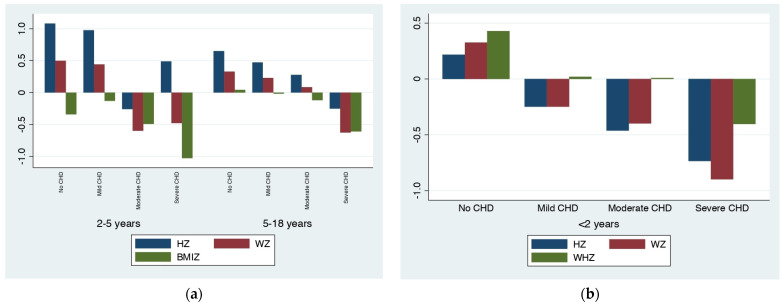
(**a**) Graphical distribution of mean length/height-for-age z-score (HZ), weight-for-age z-score (WZ) and Body-Mass-Index-for-age z-score (BMIZ)according to Congenital Heart Disease (CHD) severity in children older than 2 years old, divided into age groups; (**b**) Graphical distribution of mean weight-for-height z-score (WHZ) according to CHD severity in children younger than 2 years of age.

Differences in anthropometric measures among CHD severity groups were detected, relating to the severity of the disease: the more severe the CHD, the lower the mean values. This difference was more remarkable in patients older than 5 years when considering HZ, WZ and BMIZ and in children younger than 2 years when considering WZ. Figure 1 and Figure 2 graphically show the progressive lowering of the HZ, WZ, BMIZ and WHZ distributions with increasing CHD severity.

When adjusted for low weight at birth, the association between CHD severity and lower WZ remains statistically significant (univariate model coefficient = −0.31, IC −0.43; −0.20, *p* < 0.001; post-adjustment coefficient = −0.34, IC −0.45; −0.23, *p* < 0.001).

Moreover, the association between CHD severity and lower HZ remains statistically significant when adjusted for low weight at birth (univariate model coefficient = −0.31, IC −0.43; −0.19, *p* < 0.001; post-adjustment coefficient = −0.36, IC −0.48; −0.24, *p* < 0.001).

Cyanosis was present in 31 patients, who were all members of the 88-patient severe CHD group (35%, *p* < 0.001). Adjusting for cyanosis, the association between CHD severity and lower HZ, WZ remains statistically significant (coefficient = −0.28 and −0.26, respectively, *p* < 0.001). When compared to acyanotic patients, cyanotic children suffered more often from underweight (*p* < 0.01) and stunting (*p* = 0.015), while wasting wa equally distributed (*p* = 0.394 for BMIZ, *p* = 0.06 for WHZ).

In Table 4. the frequency of surgical interventions according to CHD severity is reported. Most patients (76%) with mild CHD have not undergone surgery, mainly due to the absence of surgical indication, while some of them have undergone transcatheter correction.

Most patients (68%) with moderate CHD underwent at least one heart surgery and most patients with severe CHD (69%) had at least two surgical interventions.

Eight patients with severe CHD have not received surgery or are on the list to perform it or there is no surgery that could improve the prognosis.

## 4. Discussion

Screening growth and neurological development is a primary part of every clinical evaluation during infancy and childhood. This evaluation becomes even more important when concerning children with CHD. In fact, growth impairment is one of the most common problems among children with congenital heart disease affecting final stature and neurodevelopmental outcomes [6,37].

As reported in other European studies, [22,26,38], we observed a reduced growth in cardiopathic children compared to healthy controls, with a more negative impact as the severity of the heart disease increases. This could be explained by the increased metabolic requests of these conditions, prolonged hospitalizations and the major proportion of children with heart failure and related feeding difficulties [8]. Cyanotic patients are reported to have more episodes of wasting than their acyanotic counterparts [3,18] because of chronic hypoxia (from right-to-left shunts), polycythemia, increased metabolic stress and possible prolonged pulmonary hypertension. In our study, we did not confirm the increased prevalence of wasting in this population, but stunting and underweight were more frequent. This result could be biased by the small number of patients (5.4% of the CHD patients).

Children younger than two years of age with CHD appear more prone to poor weight gain compared to healthy controls (Table 2 and Table 3, Figure 2b). This has already been observed [2,16,38,39,40] and it is probably secondary to the significant percentage of children in this age group who have not yet performed corrective or palliative surgery and have not achieved stability of cardiac hemodynamics. In fact, delay in the surgical correction of heart defects is known to be associated with undernutrition [1] and poor growth results, especially in children less than five years of age.

At our center, surgical intervention is usually performed early in life and this could explain the generally small percentages of stunting, wasting and underweight that were observed, with catch-up growth being seen later in life (Figure 1a,b and Figure 2) in accordance with what has already been reported [19,20,26,41].

In the group of patients of 5–18 years, the metabolic requests of severe heart diseases return to adversely affect growth in a relevant way. Impaired growth in this population has been related to reduced IGF-1 levels in cyanotic patients [42] and to a decreased duration and intensity of the pubertal growth spurt, resulting in a loss of height. This event is well described during puberty in chronically ill patients [43].

Many experiences from low–middle-income countries report a high prevalence (around 40%) of undernutrition in CHD patients [1,14,15,16,18], while most cardiopathic patients in our study have anthropometric parameters in the normal range, highlighting the fundamental impact of social and economic conditions—associated with precocious surgery—in the growth possibilities of children with heart disease.

At our center, nutritional rehabilitation strategies for weight gain are applied since infancy; when high-calorie formulas or drinks are recommended, daily caloric intake is increased, foods with high fat content are prescribed and, eventually, a nasogastric tube is employed. Most nutritional interventions focus on treatment strategies that prioritize adequate growth and development, independent of the type and severity of the cardiac lesion.

The percentage of underweight in our population of cardiopathic children is 6.9%, which is quite comparable to the Danish experience (9.8%) [26]. Nevertheless, the general prevalence of wasting among German children under the age of 5 years is reported to be 0.3% [44] and the percentages of stunting and underweight in German children under 5 years of age are 1.6% and 0.5%, respectively [45,46] (no general data about Italian children have been collected so far). Therefore, even if treated early in life, heart disease remains significant in impacting the growth of children in European countries.

Overweight and obesity are growing problems in high-income countries: the Global Nutrition Report 2020 states a prevalence of obesity in children and adolescents (5–19 years) of 11.1% for girls and 15.2% for boys [47]. In the United States, a prevalence of obesity of 12–15% was documented among children with CHD, similar to the 16% of all children in the same age group presenting obesity [31,34]. The prevalence of obesity was lower in CHD patients from Denmark and Switzerland (2.3% and 6.7%) [22,26] and similar to the general population. In our study, the overall prevalence rate of obesity is low (2.3%) and comparable between children with and without congenital heart disease and similar to the European data. Thus, children with congenital heart disease are not safe from the obesity epidemic and the associated cardiovascular risk factors in adulthood. As expected, the prevalence of obesity among patients with the most complex forms of CHD is lower, which may be explained by the high metabolic demand, ongoing heart failure or cyanotic disease.

As expected, the more severe the CHD, the greater the number of surgical interventions for each patient. Most patients (68%) with moderate CHD underwent at least one heart surgery in the pediatric age and most patients with severe CHD (69%) had at least two surgeries. In our experience, most CHD patients with mild lesions required one-time (22%) or no surgical intervention (76%). This happened because mild lesions do not often present surgical indication, they may resolve with time, or they can be corrected by percutaneous procedure. In our study, patients with mild CHD presented anthropometric parameters similar to those of healthy children (Table 1 and Table 3, Figure 1 and Figure 2a), probably due to the reduced hemodynamic relevance of these types of lesions (mild isolated pulmonary stenosis, ASD and VSD with mild shunt, small PDA and mild aortic valve stenosis). Patients with mild lesions rarely require restrictions to activities but may carry the stigma of heart disease: not being encouraged to participate in physical activities that ‘‘stress the heart’’ and adopting a relatively sedentary lifestyle, which can lead to an increased risk of obesity [48]. Previous studies have noted that pediatric CHD patients and their parents are often misinformed regarding activity restrictions [49,50]. Therefore, it is important to perform proper family counselling: informing patients of the benefits of physical activity and the additional cardiovascular risk associated with obesity and suggesting nutritional aid, when necessary.

In our unit, we usually counsel patients on diet and exercise during routine visits. This, together with the Mediterranean diet [51], could explain the low percentage of obese patients observed in our study.

## 5. Conclusions

Moderate and severe congenital heart diseases negatively affect the growth of children compared to general population even in a center from a high-income country, where surgery is performed early and nutritional support is guaranteed. This is particularly noticed in the first two years of life and late in adolescence.

Most CHD patients have anthropometric parameters in the normal range and the percentage of undernourished patients is higher compared to healthy children, especially in the group of patients with severe congenital heart disease. Moreover, children with congenital heart diseases are not safe from the obesity epidemic, with a percentage of obese children comparable to that of the general population. Therefore, we believe that the management of nutritional status in cardiopathic patients can still be improved by exposing patients to early nutritional counselling and organizing periodic nutritional status assessments in a competent setting to avoid undernutrition and obesity, especially in patients with severe heart diseases.

## 6. Study Limitations

This was a retrospective monocentric study with a relatively small number of participants for each group of patients (especially healthy children and children with severe CHD) and a significant different mean age between healthy and CHD children.Data about physical activity were incomplete; therefore, these data were not reported. The population was not characterized by ethnicity or economic background, factors that are known to influence the growth and the obesity prevalence in the general population. To better characterize the impact of surgery on patients’ growth, longitudinal anthropometric data for each patient should be collected and a larger study population would allow more clinically relevant analyses on subgroups. Future investigation should also be focused on the evaluation of the neurological development and cognitive function of CHD patients stratified for disease severity.

## Figures and Tables

**Table 1:** HZ, WZ, BMIZ and WHZ in CHD severity groups. The frequency ofwasting underweight, stunting, overweight, obesity and tall stature in the different groups is reported.

*CHD	Absent (*n* = 91)	Mild (*n* = 255)	Moderate (*n* = 223)	Severe (*n* = 88)	Tot (*n* = 657)	*p*-Value
^∆^HZ, mean ± SD	0.62 ± 1.2	0.38 ± 1.37	0.04 ± 1.39 **	−0.29 ± 1.48 **	0.21 ± 1.39	**<0.001**
°WZ, mean ± SD	0.37 ± 1.01	0.16 ± 1.23	−0.09 ± 1.30 **	−0.67 ± 1.41 **	−0.01 ± 1.29	**<0.001**
^¥^BMIZ, mean ± SD (≥2 Ys)	−0.07 ± 1.13	−0.03 ± 1.24	−0.16 ± 1.29	−0.65 ± 1.3 **	−0.19 ± 1.42	**0.004**
^§^WHZ, mean ± SD	0.43 ± 1.07	0.02 ± 1.45	0.01 ± 0.1.42 **	−0.40 ± 1.71	0.04 ± 1.4	**0.009**
^∆^HZ < −2 SD, N (%)	1 (1.1%)	8 (3.1%)	20 (8.9%) **	9 (12.3%) **	38 (5.8%)	**0.003**
°WZ < −2 SD, N (%)	0 (0%)	8 (3.1%)	16 (7.1%)	15 (17%) **	39 (5.9%)	**<0.001**
^¥^BMIZ < −2 SD, N (%) (≥2 Ys)	2 (2.2%)	8 (3.8%)	8 (4.9%)	11 (15.7%) **	29 (4.4%)	**0.001**
^§^WHZ < −2 SD, N (%) (<2 Ys)	0 (0%)	4 (8.8%)	7 (11.9%)	4 (22.2%)	15 (10.1%)	0.107
^∆^HZ > 2 SD, N (%)	12 (13%)	35 (13.7%)	11 (4.9%) **	2 (2.3%) **	60 (9.1%)	**<0.001**
°WZ > 2 SD, N (%)	6 (6%)	16 (6.3%)	12 (5.4%)	3 (3.4%)	37 (5.6%)	0.757
^¥^BMIZ > 2 SD, N (%) (≥2 Ys)	0 (0%)	6 (2.8%)	6 (3.6%)	0 (0%)	12 (2.3%)	0.193
^§^WHZ > 3 SD, N (%) (<2 Ys)	0 (0%)	1 (2.2%)	0 (0%)	0 (0%)	1 (0.6%)	0.512

^¥^BMIZ: Body-Mass-Index-for-age z-score, *CHD: Congenital Heart Disease, ^∆^HZ: length/height-for-age z-score, ^§^WHZ: weight-for-height z-score, °WZ: weight-for-age z-score. ** significant differences according to Bonferroni analysis. In bold significant *p*-values are highlighted (*p*-value < 0.05)

**Table 2:** HZ, WZ, BMIZ and WHZ differences between healthy children and children with CHD stratified for age groups.

*CHD		Absent (*n* = 91)	CHD (*n* = 566)	*p*-Value
^∆^HZ, mean ± SD	<2 years (*n* = 148)	0.22 ± 1.39	−0.42 ± 1.82	0.0926
	2–5 years (*n* = 61)	1.08 ± 0.88	0.37 ± 1.12	**0.0194**
	≥5–18 years (*n* = 448)	0.66 ± 1.16	0.29 ± 1.25	0.0502
°WZ, mean ± SD	<2 years (*n* = 148)	0.03 ± 1.05	−0.42 ± 1.54	**0.0201**
	2–5 years (*n* = 61)	0.49 ± 1.12	−0.16 ± 1.21	**0.0499**
	≥5–18 years (*n* = 448)	0.23 ± 1.18	0.04 ± 1.23	0.3360
^§^WHZ, mean ± SD	<2 years (*n* = 148)	0.43 ± 1.07	−0.05 ± 1.47	0.1187
^¥^BMIZ, mean ± SD	2–5 years (*n* = 61)	−0.34 ± 1.32	−0.43 ± 1.09	0.7746
	≥5–18 years (*n* = 448)	−0.27 ± 2.37	−0.15 ± 1.30	0.5845

^¥^BMIZ: Body-Mass-Index-for-age z-score, *CHD: Congenital Heart Disease, ^∆^HZ: length/height-for-age z-score, ^§^WHZ: weight-for-height z-score, °WZ: weight-for-age z-score. In bold significant *p*-values are highlighted (*p*-value < 0.05)

**Table 3:** HZ, WZ, BMIZ and WHZ divided for age groups and stratified for CHD severity.

*CHD	Age Groups	Absent (*n* = 91)	Mild (*n* = 255)	Moderate (*n* = 223)	Severe (*n* = 88)	*p*-Value	*p*-Value Bonferroni Analysis
^∆^HZ, mean ± SD	≤2 years (*n* = 148)	0.22 ± 1.39	−0.25 ± 1.91	−0.46 ± 1.81	−0.74 ± 1.67	0.279	
	2–5 years (*n* = 61)	1.08 ± 0.88	0.97 ± 1.06	−0.26 ± 1.13	0.48 ± 0.45	**0.0005**	Moderate vs. Mild or Absent 0.001 and 0.003
	>5 years (*n* = 448)	0.65 ± 1.16	0.47 ± 1.19	0.27 ± 1.14	−0.25 ± 1.47	**0.0002**	Severe vs. other groups 0.01, <0.001 and 0.25
°WZ, mean ± SD	≤2 years (*n* = 148)	0.33 ± 1.05	−0.25 ± 1.53	−0.39 ± 1.45	−0.89 ± 1.77	**0.048**	Severe vs. Absent 0.042
	2–5 years (*n* = 61)	0.49 ± 1.12	0.44 ± 1.22	−0.59 ± 1.12	−0.48 ± 0.91	**0.0097**	Moderate vs. Mild or Absent 0.027 and 0.051
	>5 years (*n* = 448)	0.33 ± 0.96	0.23 ± 1.13	0.08 ± 1.22	−0.63 ± 1.36	**<0.001**	Severe vs. other groups < 0.001
^§^WHZ, mean ± SD	≤2 years (*n* = 148)	0.43 ± 1.07	0.02 ± 1.45	0.01 ± 1.41	−0.40 ± 1.71	0.289	
^¥^BMIZ, mean ± SD	2–5 years (*n* = 61)	−0.34 ± 1.32	−0.13 ± 1.11	−0.49 ± 1.05	−1.03 ± 1.04	0.379	
	>5 years (*n* = 448)	0.04 ± 1.03	−0.02 ± 1.26	−0.12 ± 1.32	−0.61 ± 1.33	**0.010**	Severe vs. absent or mild 0.049 and 0.008

^¥^BMIZ: Body-Mass-Index-for-age z-score, *CHD: Congenital Heart Disease, ^∆^HZ: length/height-for-age z-score, ^§^WHZ: weight-for-height z-score, °WZ: weight-for-age z-score. In bold significant *p*-values are highlighted (*p*-value < 0.05)

**Table 4:** Surgical interventions according to CHD severity groups.

	N° Surgery	No Surgery	One Surgical Procedure	Two Surgical Procedures	Three Surgical Procedures	Four Surgical Procedures	Five Surgical Procedures
CHD Severity							
Mild CHD (255 patients)		194 (76%)	55 (21.6%)	5 (2%)	1 (0.4%)	0	0
Moderate CHD (223 patients)		71 (31.8%)	117 (52.4%)	27 (12.1%)	6 (2.7%)	2 (0.9%)	0
Severe CHD (88 patients)		8 (9.1%)	19 (21.6%)	36 (40.9%)	24 (27.3%)	0	1 (1.1%)

## Data Availability

The data presented in this study are available on request from the corresponding author. The data are not publicly available due to privacy and ethical restrictions.
